# Mechanical Activation of Adipose Tissue and Derived Mesenchymal Stem Cells: Novel Anti-Inflammatory Properties

**DOI:** 10.3390/ijms19010267

**Published:** 2018-01-16

**Authors:** Stephana Carelli, Mattia Colli, Valeriano Vinci, Fabio Caviggioli, Marco Klinger, Alfredo Gorio

**Affiliations:** 1 Pediatric Clinical Research Center “Fondazione Romeo e Enrica Invernizzi”, University of Milan, 20142 Milan, Italy; stephana.carelli@guest.unimi.it (S.C.); mattiacolli@live.it (M.C.); 2 Humanitas Research Hospital, Plastic Surgery Unit, Via Manzoni 56, 20089 Rozzano, Italy; valeriano.vinci@humanitas.it (V.V.); Marco.klinger@humanitas.it (M.K.); 3 Multimedica San Giuseppe Hospital, Plastic Surgery Unit, Via San Vittore 12, 20123 Milan, Italy; fabio.caviggioli@gmail.com; 4 Laboratory of Pharmacology, Department of Health Sciences, University of Milan, Via A. di Rudinì 8, 20142 Milan, Italy

**Keywords:** adipose tissue, mechanical activation, gene activation, mesenchymal stem cells, inflammation

## Abstract

The adipose tissue is a source of inflammatory proteins, such as TNF, IL-6, and CXCL8. Most of their production occurs in macrophages that act as scavengers of dying adipocytes. The application of an orbital mechanical force for 6–10 min at 97 g to the adipose tissue, lipoaspirated and treated according to Coleman procedures, abolishes the expression of TNF-α and stimulates the expression of the anti-inflammatory protein TNF-stimulated gene-6 (TSG-6). This protein had protective and anti-inflammatory effects when applied to animal models of rheumatic diseases. We examined biopsy, lipoaspirate, and mechanically activated fat and observed that in addition to the increased TSG-6, Sox2, Nanog, and Oct4 were also strongly augmented by mechanical activation, suggesting an effect on stromal cell stemness. Human adipose tissue-derived mesenchymal stem cells (hADSCs), produced from activated fat, grow and differentiate normally with proper cell surface markers and chromosomal integrity, but their anti-inflammatory action is far superior compared to those mesenchymal stem cells (MSCs) obtained from lipoaspirate. The expression and release of inflammatory cytokines from THP-1 cells was totally abolished in mechanically activated adipose tissue-derived hADSCs. In conclusion, we report that the orbital shaking of adipose tissue enhances its anti-inflammatory properties, and derived MSCs maintain such enhanced activity.

## 1. Introduction

Several agents involved in inflammatory processes are made by the adipose tissue. Compared with leaner individuals, the adipose tissue of obese persons shows higher expression of pro-inflammatory proteins, such as TNF-α, interleukin 6 (IL-6), monocyte chemotactic protein 1, transforming growth factor β1, pro-coagulant proteins, such as plasminogen activator inhibitor type 1, tissue factor, and factor VII [1,2,3,4,5,6,7,8,9,10]. Also, the macrophage number increases in adipose tissue of obese individuals [11,12], where they apparently function to scavenge moribund adipocytes, whose number increases dramatically with obesity [13]. Macrophages are likely the source of most cytokine production in adipose tissue of obese people [10,11,12]. In fact, macrophages resident in adipose tissue are responsible of the production of almost all adipose tissue TNF-α, release high amounts of IL-6, and produce an inducible isoform of nitric oxide synthase [11]. In particular, Xu et al. [12] reported that in adipose tissue of obese mice the expression of inflammation-specific genes by macrophages was increased. This is preceded by an intense augmentation of insulin production. Furthermore, when rosiglitazone, an insulin-sensitizing drug, was administered to those mice the expression of the above-reported genes and others decreased. Thus, the appropriate sequence of appearance of inflammatory molecules before the development of insulin resistance, as well as their known ability to promote both insulin resistance and other complications of obesity, strongly suggests adipose tissue inflammation as an important player in the development of obesity-related complications. Here, we report that the application of a mechanical force for a few minutes abolishes the inflammatory properties of fat and favors the expression of anti-inflammatory proteins, such as TNF-stimulated gene-6 (TSG-6). This anti-inflammatory cytokine has been shown to exert protective effects when administered in disease experimental models and through the reduction of infiltrating neutrophils and neutralizing CXCL8 [14]. This may render the use of fat tissue in human clinical transplantation procedures much more reliable and prone to a positive outcome.

Mesenchymal stem cells (MSCs) are defined as multi-potent, self-renewing cells with the ability to differentiate, in vitro, into cells of mesenchymal origin, such as osteoblasts, adipocytes, and chondrocytes, and give rise to bone, fat, cartilage, and muscle tissues in vivo [15]. Moreover, many studies have shown that the potentiality of tissue regeneration can be enhanced by using adipose-derived stem cells (ADSCs) (see revision in ref. [16]). In fact, by using a colony-forming unit-fibroblasts (CFU-F) assay it has been shown that stem cells frequency is significantly higher in adipose tissue than in other tissues, such as bone marrow [17]. Indeed, the autologous transplant of adipose tissue is an established therapeutic procedure used for the repair of a variety of tissue damage. To minimize discrepancies and inconsistencies, and allow for the comparison of data generated in different laboratories, the International Society of Cellular Therapy (ISCT) has provided guidelines for the definition of mesenchymal stem cells (MSCs) based on their (a) plastic adhesion proprieties; (b) immune-phenotype; and (c) multi-potent differentiation potential [18,19]. Clinical and pre-clinical studies show that autologous MSCs survive after transplantation, show pluripotential differentiation [20,21,22,23], and exert anti-apoptotic, anti-inflammatory, and angiogenic effects [24,25,26,27,28]. Here, we show that human adipose tissue-derived mesenchymal stem cells (hADSCs) derived from mechanically activated adipose tissue grow well in culture, as they maintain their karyotype, their differentiation is normal, and most of all these cells show much higher anti-inflammatory properties than control hADSCs from mechanically unstimulated adipose tissue.

Mechanical forces may be of key relevance during embryogenesis and tissue remodeling and for the mitotic and motile capabilities of cells [29]. This suggested us the hypothesis that under the mechanical manipulation of fat tissue, cells perceive and respond to externally applied mechanical forces and forces exerted by the cell matrix and cell–cell contacts. This causes the activation of signals, such as stretch-activated ion channels, growth factors, cytoskeleton filaments, extracellular matrices, focal adhesions, and tyrosine kinases, that appear to be involved in the interpretation of mechanical forces into biochemical events within the cells (mechano-transduction), driving downstream signals that communicate key messages to the nucleus [30,31,32,33,34]. Furthermore, mechano-transduction has been postulated to play an important role in tissue engineering applications and regenerative medicine strategies [30,35].

## 2. Results

### 2.1. Tissue Mechanical Activation

Human adipose tissue samples have been obtained from elective liposuction procedures under local anesthesia. 

**Figure 1:**
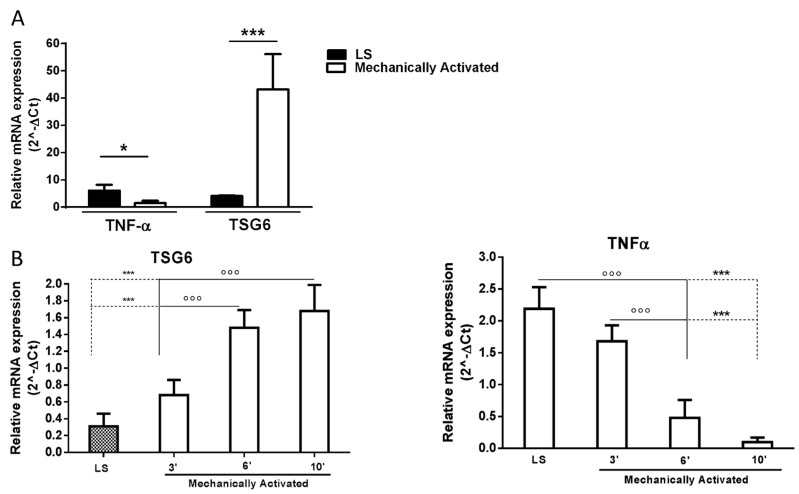

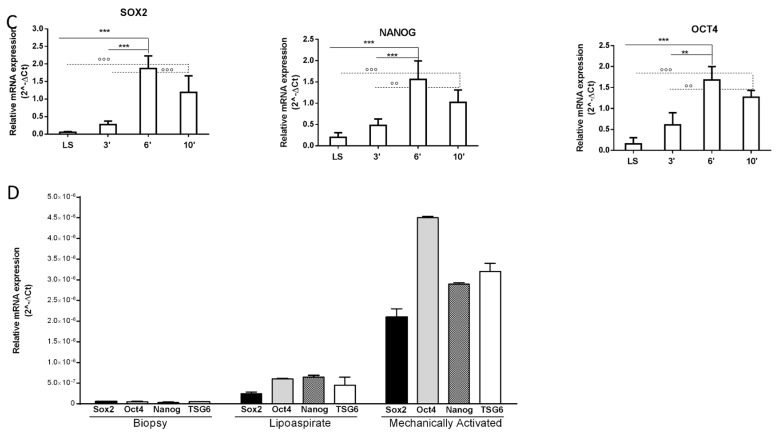
Differential expression of cytokines and pluripotency genes in lipoaspirated adipose tissue after mechanical activation. Expression levels of cytokine mRNAs (TSG6 and TNF-α) and pluripotency genes (Sox2, Nanog, and Oct4) were investigated by real-time RT-PCR on total RNAs extracts of lipoaspirated fat that was mechanically treated (MA) with the application of an orbital shaking force (97× *g*) for different time points (3, 6, and 10 min) or not (LS). The assay was performed on all investigated samples with similar results. Data represented in the graphs refer to at least six patients per point (**A**,**C**) and four different samples (**B**). Each sample was evaluated in triplicate and data are reported as mean ± SD. In (**A**) * *p* < 0.5, *** *p* < 0.001 vs. LS; in (**B**) *** *p* < 0.001 vs. LS and °°° *p* < 0.001 vs. mechanically activated for 3 min. In (**C**) °°° *p* < 0.001 vs. LS and °° *p* < 0.01 vs. mechanically activated for 3 min, *** *p* < 0.001 vs. LS, *** *p* < 0.001, ** *p* < 0.01 vs. mechanically activated for 3 min. In (**D**), it is shown the data obtained from the three original biopsies, which subsequently were lipoaspirated and further activated mechanically.

Ten ml of washed lipoaspirated adipose tissue was place in a 50 mL conical tube and activated by orbital shaking (97× *g*) for different times (3, 6, and 10 min) at room temperature. Messenger RNAs of pro- and anti-inflammatory factors were assessed in lipoaspirated tissue subjected to mechanical forces for 10 min. The untreated lipoaspirate acted as control. The results show that the application of a 97× *g* force modified the typical expression of the inflammatory cytokine tumor necrosis factor-α (TNF-α), which was drastically reduced and significantly downregulated compared to the control (untreated lipoaspirate). Differently, the expression of its inhibitor TSG6 markedly and significantly increased (Figure 1A). The data were obtained from fat donated by six patients. The process of the mechanical activation of anti-inflammatory markers is time-dependent, as shown in Figure 1B. Conversely, TNF-α production was completely inhibited within 6 min under the same conditions (Figure 1B).

Nanog, Oct4, and Sox2 are three transcription factors expressed at high levels in embryonic stem cells. These factors regulate the activation or repression of other genes during development and are found expressed at high levels in pluripotent cells of the inner cell mass. The downregulation of these three transcription factors correlates with the loss of pluripotency and self-renewal [36]. These genes are expressed in some MSCs, such as breast milk stem cells [36], bone marrow stem cells [37], and term amniotic fluid stem cells [38]. The pluripotency regulatory genes Sox2, Nanog, and Oct4 are fully activated within 6 min of 97× *g* mechanical activation (Figure 1C). Thus, in addition to the anti-inflammatory properties, the parameters defining the stemness of cells are also increased by the applied mechanical stress in a force- and time-dependent manner (Figure 1C).

The level of activation of such stemness genes and TSG-6 was minimal in normal biopsy fat tissue and was slightly enhanced by classic liposuction manipulation done according to Coleman’s procedure. However, the induction of their activation was markedly higher when the mechanical procedure was applied (Figure 1D).

### 2.2. Preparation of Mesenchymal Stem Cells Cultures

Starting from adipose tissue subjected to a 97× *g* force, we were able to isolate and expand hADSCs through reproducible methods recently described [39]. Two mL of lipoaspirated adipose tissue mechanically treated with a 97× *g* force were placed in 25-cm^2^ culture flasks with 5 mL of growth medium (alpha MEM + 10% FBS). This allows the tissue to adhere to the floor of the plate. After 2 weeks in culture, the adipose tissue was removed, and cells were maintained in culture. After 20 days, the cells reached 90% confluence. Starting from each plate containing 2 mL of mechanically treated adipose tissue, the yield of cells is about 5.5–6 × 10^5^ cells. 

**Figure 2:**
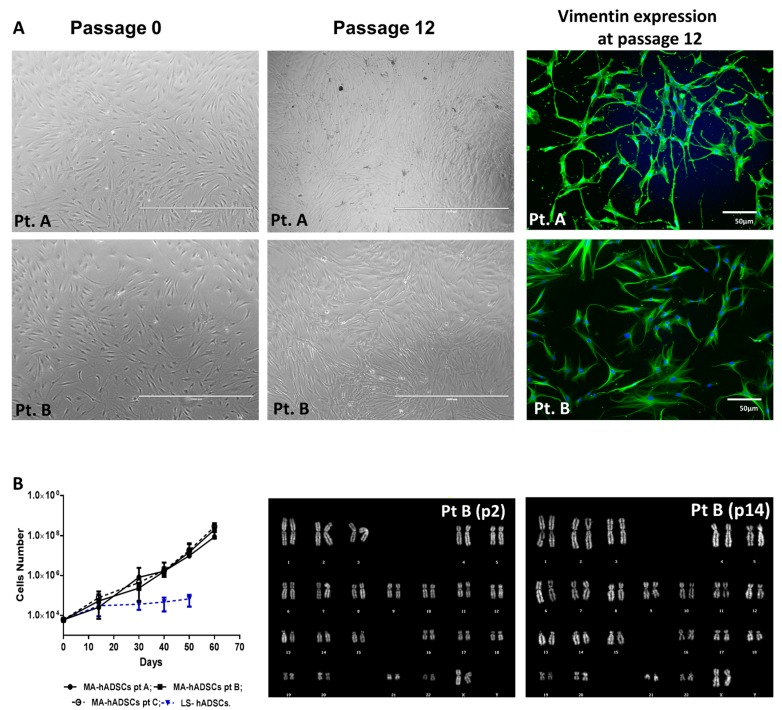
In vitro characterization of human adipose tissue-derived mesenchymal stem cell (hADSC) cultures. (**A**) Live morphology of hADSCs obtained from adipose tissue treated with an orbital shaking force (10 min). Pictures refer to two different cases (Pt. A and Pt. B) that were kept at two different passages in culture (passage 2 and passage 12, respectively). The expression of the mesenchymal marker vimentin was evidenced by immunofluorescence and is shown in green. Nuclei were stained with DAPI in blue; (**B**) Expansion curves of hADSCs derived from activated adipose tissue. Each point of the growth curves represent the mean ± standard error of the mean (SEM) of counts performed in triplicate for cells obtained from three different cases (Pt. A, Pt. B, and Pt. C). The growth curve of hADSCs derived from one LS sample was inserted into the graph as control. Even in this case, counts were performed in triplicate at each time point and are reported as mean ± SEM. Karyotype analyses were performed on an early (passage 2) and a late passage (passage 14) for hADSCs maintained in αMEM supplemented with 15% FBS and antibiotics. These results are representative of the same analyses performed in the other ten cases.

The live morphology of these cells shows a fibroblast-like phenotype comparable to that of cells obtained with classical methods (Figure 2A). Approximately 100% of hADSCs obtained from activated fat expressed the mesenchymal marker vimentin (Figure 2A). To investigate the growth capability of purified hADSCs obtained from activated fat, expansion curves of cell populations obtained from three different cases (Figure 2B) were established. The cell doublings of the hADSC cultures were comparable among the different cases at the same time points (Figure 2B). Mechanically activated fat-derived hADSCs maintained in culture did not present any chromosomal rearrangement, as assessed by QFQ banding performed at early and late passages (Figure 2B). The chromosome number was normal in all analyzed samples of hADSCs derived from mechanically activated fat (*n* = 3) [39].

**Figure 3:**
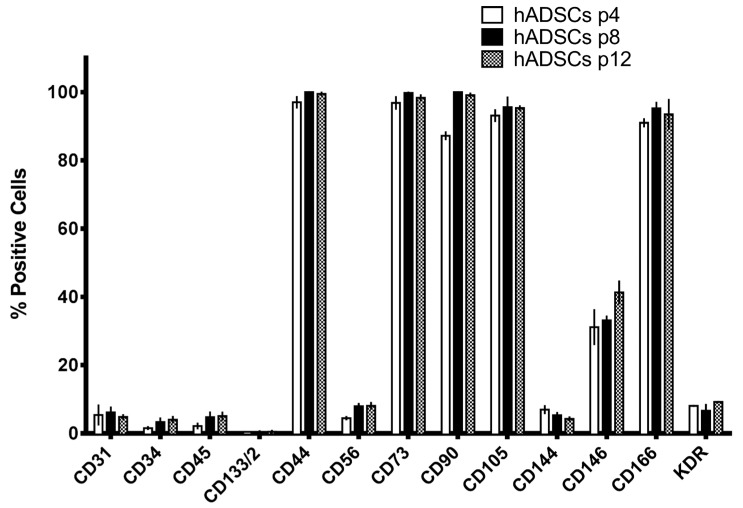
Fluorescence-Activated Cell Sorting (FACs) analysis of hADSCs isolated from activated lipoaspirates. Cell surface phenotype of hADSCs derived from lipoaspirated adipose tissue activated with the application of an orbital shaking force (97× *g*) for 10 min. Cell surface phenotype was investigated by FACS (see Materials and Methods for details) on hADSCs maintained in αMEM at three different passages in culture (passage 2, white bars; passage 8, black bars; passage 12, grey bars). The assay was performed in three different cases, and each case at each passage was investigated in triplicate. Mean ± SEM of the three different cases are reported in the graphs.

The hADSCs isolated with the above-described protocol were characterized for the expression of mesenchymal markers by means of a FACS analysis (Figure 3). These hADSCs express at high values (about 90–100%) known mesenchymal markers (such as CD44, CD73, CD90, and CD166) (Figure 3). Some hematopoietic markers (such as CD45, CD133, and CD56) are also represented (Figure 3), while endothelial markers (such as CD31, CD34, CD144, CD146, and KDR) are more highly represented in cells obtained from 97× *g* treated adipose tissue (Figure 3). Moreover, the profile of surface antigen expression was maintained among different passages in culture (Figure 3).

### 2.3. Adipogenic and Osteogenic Differentiation

**Figure 4:**
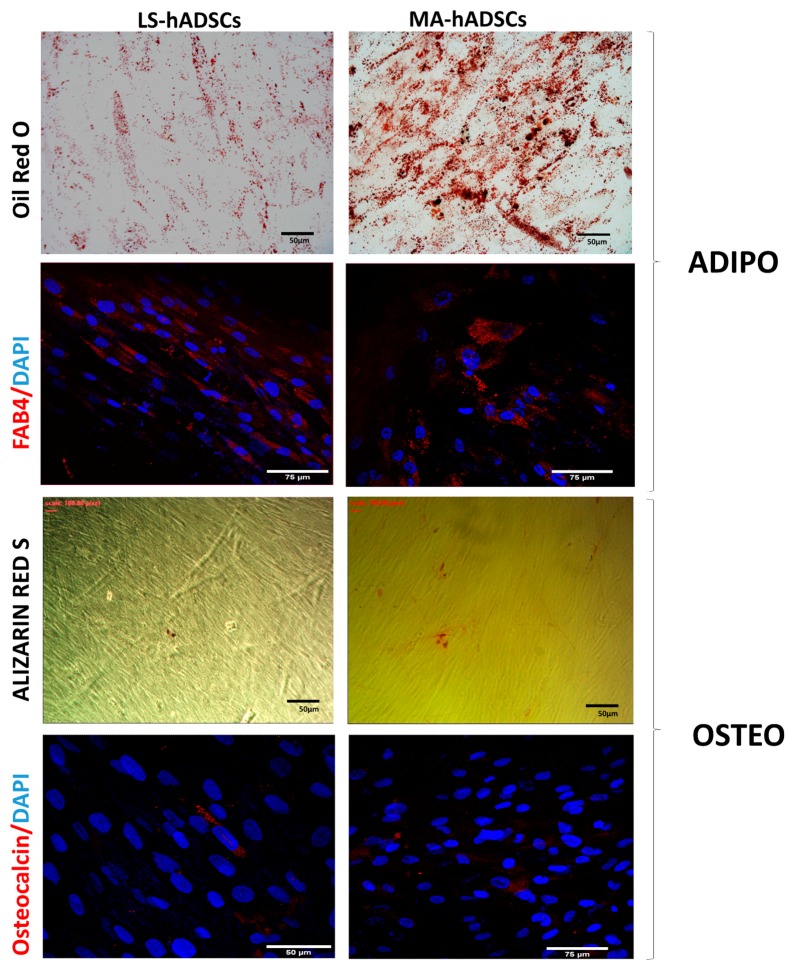
hADSC in vitro differentiation. Cells obtained from lipoaspirated adipose tissue mechanically activated (MA-hADSCs) or not (LS) were differentiated in vitro (as described in the Materials and Methods section). Adipogenesis was revealed by Oil red O staining for lipid droplets and with the positivity to the adipogenic marker FABP4 evidenced with immunofluorescence. FABP4 is shown in red and nuclei in blue (DAPI). Osteogenic differentiation was marked by the formation of mineralized matrices as shown by Alizarin red staining and osteocalcin expression by immunostaining. Osteocalcin is shown in red and nuclei in blue (DAPI). Data are representative of two different experiments for four cases for MA-hADSCs and two cases for LS.

The differentiation ability of mechanically activated, fat-derived hADSCs was investigated in vitro, and differentiation experiments were set up for the evaluation of the mesenchymal commitment. By using a 21 day-long classical adipogenic differentiation approach [39], it was observed that after adipogenic induction hADSCs accumulated small bubble-shaped Oil red *O*-positive lipid droplets in their cytoplasms (Figure 4). This evidence was corroborated also by the positive staining with anti-fatty acid binding protein 4 (FABP4), a marker of mature adipocytes, at day 21 of differentiation (Figure 4) [40]. In the presence of osteogenic medium, cultures of hADSCs derived from fat activated with a 97× *g* force reached almost complete confluence within a week of seeding. Later, cells formed aggregates characterized by the presence of amorphous material deposits, which were positive by Alizarin red S staining (Figure 4). The osteogenic differentiation capability was confirmed by investigating the expression of osteocalcin, an osteoblast-specific hormone (Figure 4) [41].

### 2.4. hADSCs Isolated from Mechanically Activated Adipose Tissue Show Strong Anti-Inflammatory Features

**Figure 5:**
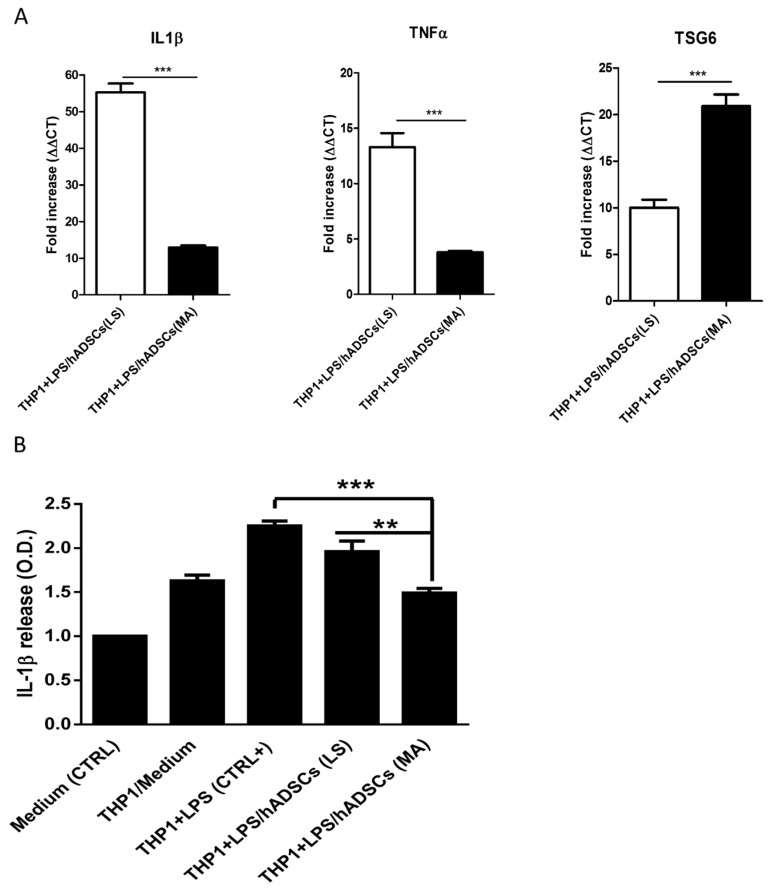
Anti-inflammatory action of isolated hADSCs from mechanically activated adipose tissue. (**A**) mRNA expression levels of pro-(IL-1β and TNF-α) and anti-(TSG6) inflammatory cytokines were investigated by real time RT-PCR on total RNAs extracts of activated THP1 cells (see Materials and Methods for details) after 3-h treatments with hADSCs obtained from LS or the same LS mechanically activated with orbital shaking (97× *g*) for 10 min. Each experiment was repeated in duplicate and each sample was investigated in triplicate. Data are reported as mean ± SEM; (**B**) IL-1β release by THP1 cells in the medium. IL-1β was determined by slot blot of 1 mL of THP-1-activated cells in reported conditions. Bands intensity was quantified in triplicate by using KODAK 440 and reported as mean optical density (O.D.) ± SD. ** *p* < 0.01; *** *p* < 0.001.

It is well-known that hADSCs are able to affect inflammation [42]. THP-1 cells are a well-known model for studying monocytes’ and macrophages’ functions [43]. Interleukin-1β (IL-1β) is a potent pro-inflammatory cytokine released by many cell types, but it mainly is investigated as released by monocytes and macrophages [44]. The stimulation of monocytes with the bacterial endotoxin lipopolysaccharide (LPS) causes an increase in IL-1β production and release in the culture medium [45]. The anti-inflammatory action of hADSCs obtained from mechanically activated adipose tissue was evaluated in co-culture assays (trans-well, see experimental procedure) with THP1 cells activated with LPS (10 microgr/mL for 3 h). By real time RT-PCR, we investigated the messenger RNA’s expression of both pro-inflammatory (IL-1β and TNF-α) and anti-inflammatory cytokines (TSG6) in THP1-cells. As shown in Figure 5A, the expression of IL-1β was much more significantly decreased when activated monocytes (THP1 + LPS) were in the presence of hADSCs obtained from 97× *g*-activated adipose tissue compared to hADSCs obtained from lipoaspirate (Figure 5A). In addition, the expression of TNF-α was also more efficiently counteracted, while TSG6 expression was higher when THP1 cells were exposed to hADSCs from mechanically activated fat tissue (Figure 5A). The THP-1 release of IL-1β in the medium was assayed by immunoblotting. These cells release in the medium a certain amount of IL-1β, which was increased by about 50% when stimulation for 3 h with LPS was applied. Such a significant increase of IL-1β release was unaffected by control hADSCs from unstimulated fat, however, when hADSCs obtained from 97× *g*-activated adipose tissue were added, the release of IL-1β was significantly reduced and practically normalized to the level measured in the absence of LPS (Figure 5B). Thus, hADSCs from mechanically activated fat inhibit the expression of inflammatory cytokines and abolish their release.

## 3. Discussion

This study reports a novel finding regarding the human adipose tissue and its related mesenchymal stromal stem cells. It is well-known that adipose tissue is a factory for inflammatory compounds, particularly when it is involved in the establishment of obesity [1,8,10]. In this paper, we have investigated the effect of a 97× *g* force, which gives rise to most consistent and repeatable results. Most of the data reported in the paper are relative to hADSCs isolated from adipose tissue activated for 10 min even if, in terms of genes activation (*TSG6*; *Sox2*; *Oct4*, and *Nanog* induction and *TNF-α* downregulation), either 6 or 10 min gave comparative results. The simple mechanical treatment, obtained by orbital shaking at 97× *g* for 6 min, allows for the development of high anti-inflammatory properties and the suppression of inflammatory agents produced by fat. It is shown that the production of TNF-α is suppressed, while that of TSG-6 is stimulated. This latter agent has potent anti-inflammatory properties and was found originally in the synovial fluids of an inflamed knee [46]. Such activity becomes more evident in hADSCs derived from activated adipose tissue; the well-known anti-inflammatory properties of hADSCs are markedly enhanced by the mechanical activation of the fat source of hADSCs. After LPS inflammation activation, there is a powerful and higher suppression of *TNF-α* and *IL-1β* coupled with a higher expression of *TSG-6*. Also, when we assayed *IL-1β* release after LPS, the activated MSCs totally eliminate the secretion of *IL-1β*.

It is very interesting to note that, looking at Figure 1D, one may notice that while the biopsy shows only a minimal trace of *Sox2*, *Oct4*, *Nanog*, and *TSG-6*, the simple Coleman treatment of the fat produced only a slight increase in these agents’ production, which is strongly activated by the orbital shaking. It is possible that adipose and stromal cells possess tension-sensitive organelles that may perceive orbital shaking and their activation may promote such anti-inflammatory properties that are memorized in their genetic code, and when hADSCs are put in culture their anti-inflammatory activity is much stronger than that of control hADSCs isolated from lipoaspirated fat. The THP-1 experiments are a clear-cut demonstration of such potentiated anti-inflammatory properties. *Sox2* has an important role in the maintenance of embryonic and neural stem cells’ pluripotency [47,48]. Furthermore, Go et al., 2008, reported that Sox2 is essential for embryonic stem cells’ pluripotency and self-renewal [49]. The same authors reported also that Sox2 is produced by somatic stem cells that have a high expansion and differentiation potential [49]. Sox2 is a transcription factor (34 Kda) that contains a specific domain to bind DNA (HMG box) and give rise to molecular complexes with Oct4 to control the expression of a number of genes. Moreover, it has also been reported that the Chip-seq on a Sox2 immuno-precipitate shows a highly efficient binding of Sox2 to the IL6 promoter in glioblastoma or ES cells [50]. The finding that mechanical activation of adipose tissue strongly enhances *Sox2*, *Oct 4*, and *Nanog* within 6 min suggests that the action of orbital shaking spreads from macrophages to the stromal cells and that such action is then maintained when we grow these cells in culture as shown by the much more efficient anti-inflammatory action exerted by hADSCs purified from the activated fat onto THP-1 cells activated by LPS. There is an inhibition of the synthesis and release of *IL-1β* by THP-1 cells.

It has been shown that TNF-stimulated gene/protein 6 (TSG-6) is a protein associated to inflammation that is upregulated by chemokines, such as IL-1, TNF, and LPS, in several types of cells [14]. It was found at high levels, originally, in the joints of patients affected by rheumatoid- and osteo-arthritis, thus it was mistaken for an inflammatory chemokine [50]; however, its administration in models of inflammatory diseases inhibited damage, reduced arthritis, and counteracted the rejection of transplants [51,52,53]. These data show that TSG6 is a protein with anti-inflammatory properties. It has also been observed that TSG-6 is expressed in human mesenchymal stem cells, where it exerts powerful anti-inflammatory effects as shown in myocardial infarction, peritonitis, traumatic brain injury, wound healing, and tissue fibrosis [14,54]. As indicated above, one of the mechanisms involved in TSG-6-mediated protective effects is its ability to abolish the influx of neutrophils at sites of inflammation and CXCL8 presentation and transport across endothelial cells. This is likely secondary to TSG-6 inhibition of CXCL8 binding to heparin at the endothelial cell surface [14]. TSG-6 binds to extracellular matrix glycosaminoglycans and inhibits the presentation of inflammatory chemokines to the cell surface, thus providing a mechanism through which the cellular presentation of chemokines is regulated.

Recently, many studies have been centered on the identification of mechanisms utilized by cells to sense and respond to mechanical stimuli (both static and dynamic) (see [55,56] for review). In response to mechanical stimuli, cells activate signaling events and remodel their intracellular architecture at both the cytoplasmic and nuclear levels [55,56]. How mechanically induced structural changes influence lineage selection in MSCs has been also investigated [57,58,59]. However, the effects of the mechanical stimulation of hADSCs have to be studied further to assess whether they have application in potential therapeutic approaches.

A key challenge in regenerative medicine is tissue minimal manipulation. Here, “minimal manipulation” has been defined as a processing approach that does not modify the original relevant characteristics of the tissue in relation to the utility of the tissue for reconstruction, repair, or replacement surgery (www.ema.europa.eu; www.fda.gov). The regulatory agencies (Food and Drug Administration and European Medicines Agency) and the tissue international society of stem cell research (ISCCR) claim that cell therapy should be considered as a medicinal product (drug) when there is more than a minimal manipulation of cells destined for application in a clinical setting or where the intended use of the cells is different to their normal function in the body (www.ema.europa.eu; www.fda.gov; [60]). The mechanical activation of adipose tissue here described follows the rule of minimal manipulation and, thus, can be highly useful in regenerative medicine for skin disease and in all of the surgical procedures where subcutaneous fat is re-introduced into the affected subcutaneous areas. In addition, the novel anti-inflammatory properties exerted against various agents may confer on the transplanted fat an extra activity that may positively influence the outcome. The reason for the reduced effect of anti-inflammatory treatments of skin diseases so far observed may be due to treatments targeting one cytokine and this may not be sufficient to reach the desired action. Differently, TSG-6 inhibits several cytokines and inhibits their binding to glycosaminoglycans, thus also abolishing cellular recruitment.

## 4. Materials and Methods

### 4.1. Tissue Mechanical Activation and Informed Consent

Lipoaspirated human adipose tissue samples were obtained from procedures of elective liposuction performed under local anesthesia with lidocaine (AstraZeneca, London, UK). This procedure involved an infiltration step, in which a solution of saline and epinephrine (2 μg/mL) (Key Customer Solutions S.A.S, Basiglio, Milan, Italy) was infused into the adipose compartment in order to minimize the loss of blood and reduce the infiltration of peripheral blood cells. The product obtained by this procedure has been termed lipoaspirate adipose tissue (Lipoaspirate).

Lipoaspirate was then activated by orbital shaking (97× *g*) for 3, 6, or 10 min at room temperature. 

Elective liposuction procedures were performed in voluntary patients with full knowledge of the events. An informed consent was supplied and signed by each single participant voluntary patient.

### 4.2. Cell Cultures

Two ml of mechanically activated adipose tissue were seeded in 25 cm^2^ flasks with 5 mL of Minimum Essential Media α medium (MEMα; Invitrogen, Carlsbad, CA, USA) supplemented with 15% fetal bovine serum (FBS; JRH Bioscience, Lenexa, KS, USA, Lot 6G2146) and incubated at 37 °C in 5% CO_2_. After 15 days, the medium and the tissue were removed and adherent cells were maintained in culture. The medium was changed every 4 days until the cells reached confluence (90–95% after about 15–20 days). To prevent spontaneous differentiation, cells were maintained at a sub-confluent culture level; therefore, when cells reached 85% confluence, they were detached from the plate by 0.05% trypsin/EDTA solution, collected by centrifugation (1200 rpm × 5 min), and expanded in culture for many passages or cryopreserved at −80 °C in the presence of dimethyl sulfoxide (DMSO; 10%).

### 4.3. Cell Growth Analysis

Analysis was performed by cell counting to determine the proliferative capacity of hADSCs obtained from the mechanically activated adipose tissue from three patients and hADSCs from the lipoaspirate from one patient, which were maintained in culture. Cells were seeded onto 48-well culture plates (Corning Life Sciences, Corning, NY, USA) and maintained in culture in growth medium. Live cells were counted by trypan blue exclusion at 85% confluence. Each point reported in the curve [36] was the mean of three determinations. We used cells obtained from fresh lipoaspirated and fresh mechanically activated adipose tissue.

### 4.4. G-Banding Karyotype Analyses

Cytogenetic studies were performed as previously described [39]. Briefly, hADSCs were directly plated onto coverslips inside Petri dishes containing 2 mL of growth medium (αMEM). As already reported by us [39], cells were then treated with Colcemid (0.02 µL/mL) (Life Technologies, Carlsbad, CA, USA) for 90 min, hypotonic solution (1:1 Na citrate 1%: NaCl 0.3%) (Sigma-Aldrich, St. Louis, MO, USA), and a fixative solution composed by methanol:acetic acid (3:1, VWR International Radnor, Radnor, PA, USA) was added and replaced twice. For karyotype analyses, at least twenty-five QFQ banding metaphases were observed for each sample [39]. The image was acquired by using a fluorescence microscope (BX 60 Olympus, Segrate, Milano, Italy) and analyzed with a Powergene PSI system (League City, TX, USA) [39].

### 4.5. Flow Cytofluorimetry: Immunophenotipic Characterization

Cultures of mechanically activated hADSCs at different passages were phenotypically characterized by Fluorescence-Activated Cell Sorting (FACS). As previously reported by us for hADScs obtained from lipoaspirate [36], after trypsinization, cells were washed with PBS and 1 × 105 cells were re-suspended in 250 uL of PBS *w*/*o* Ca^2+^ and Mg^+^ (Euroclone, Pero, Italy) and incubated with antibodies directed against specific surface markers. The following antibodies were used: anti-CD44 (BD Biosciences, Milano, Italy), anti-CD90 (Merck Millipore, Vimodrone, Milano, Italy), anti-CD34 (Mylteni Biotec, Bologna, Italy), anti-CD45 (BD Biosciences), anti-CD146 (Biocytex, Marseille, France), anti-CD31 (Mylteni Biotec), anti-CD56 (Mylteni Biotec), anti-CD105 (Biorad, Milano, Italy), anti-CD144 (R&D System, Minneapolis, MN, USA), anti-CD166 (BD Biosciences), anti-CD133/2 (Mylteni Biotec), anti-CD73 (BD Biosciences), and anti-VEGFR2 (R&D System). Samples were fixed with paraformaldehyde 4% and then studied by flow cytometry (FACS Vantage, BD Bioscience, Milano, Italy) using a specific software (CellQuest Pro, BD Bioscience). Results were expressed as mean ± SD.

### 4.6. In Vitro Differentiation

In vitro differentiation assays were performed by applying protocols previously described [39]. Briefly, for adipogenic differentiation, hADSCs from mechanically activated adipose tissue were seeded (6 × 10^3^ cells/cm^2^) in adipogenic medium [39,40] consisting of DMEM high glucose (Euroclone) supplemented with 10% FBS, 1 μmol/L dexamethasone (Sigma-Aldrich), 0.5 mM 3-isobutyl-1-methyl-xanthine (Sigma-Aldrich), and 10 μM insulin (Sigma-Aldrich). After 3 weeks in culture, cells were fixed in 10% formaldehyde for 1 h and stained with Oil red O (Sigma-Aldrich) [39,61] solution to show lipid droplet accumulation. For osteogenic differentiation, hADSCs from mechanically activated adipose tissue were seeded (9.5 × 10^3^ cells/cm^2^) in osteogenic medium containing DMEM low glucose, 10% FBS, 10 nM dexamethasone, 200 μM ascorbic acid (Sigma-Aldrich), and 10 mM b-glycerol phosphate (Sigma-Aldrich) [62]. Cells were maintained in culture for 3 weeks and then fixed in 70% ethanol and stained with 1 mg/mL Alizarin red S dye (Sigma-Aldrich) was used to detect mineralized matrices [46].

### 4.7. THP-1 Cells Culture and Activation with Lipopolysaccharides

THP-1 cells (202-TIB; ATTC, Rockville, MD, USA) were grown in suspension in RPMI 1640 media supplemented with 10% fetal calf serum (FCS), 50 U/mL penicillin, and 50 µg/mL streptomycin in 150-cm^2^ tissue culture flasks. Cell culture and all experiments were carried out at 37 °C in 5% CO_2_, 95% room air. Cells were passaged every 3 days and used between passages 3 and 12. The anti-inflammatory action of hADSCs was assayed in a trans-well culture system onto six-well plates. hADSCs were plated in the well below at the density of 1.5 × 10^4^ cells/well in alpha MEM medium. Also, Lipoaspirate Cells were used in the same conditions. Twenty-four hours after their adherence, THP-1 cells were added into the upper compartment at the density of 75 × 10^4^ cells/well. To induce IL-1β release, lipopolysaccharide (LPS) was administered at the final concentration of 10 µg/mL. After three hours of incubation, supernatants were collected in ice and immediately frozen. IL-1β release was investigated by immunoblotting.

### 4.8. Immunocytochemistry

Indirect immunofluorescence of hADSCs obtained from mechanically activated adipose tissue was performed as previously reported by us [39]. Briefly, 3.5 × 10^3^ cells/cm^2^ were seeded onto glass slides, grown until 85% confluence, and then fixed with 4% paraformaldehyde. Saturation and permeabilization was performed with 4% BSA and 0.3% Triton X-100. Then, cells were incubated for 18 h at 4 °C with primary antibodies directed against Vimentin (Polyclonal, Santa Cruz, CA, USA), FABP4 (Polyclonal, Cell Signalling, Danvers, MA, USA), and Osteocalcin (Polyclonal, Abcam, Hongkong, China). Cells were washed with PBS and then probed for 45 min with secondary antibodies Alexa Fluor 488 or 543 (Invitrogen, Carlsbad, CA, USA). Nuclei were counterstained with DAPI (2 μg/mL in PBS, Roche, Basel, Switzerland), and glasses were mounted with FluorSaveTM (Millipore, Burlington, MA, USA). Immunofluorescence was analyzed by using a Leica SP2 confocal microscope with He/Kr and Ar lasers (Heidelberg, Germany), which was also used for image acquisition. In negative controls, primary antibodies were replaced with equivalent concentrations of unrelated IgG of the same subclass [39].

### 4.9. RNA Extraction and qRT-PCR Analyses

Extractions of total RNAs were performed by using TRI Reagent^®^ (Sigma-Aldrich) and following the manufacturer’s instructions. RNA purity and quantity were determined by Nanodrop (Fisher Scientific, Hampton, NH, USA), and cDNA was obtained by retrotrascribing 1 µg of RNA with a High Capacity RNA-to-cDNA Kit (Applied Biosystems, Foster City, CA, USA).

#### 4.9.1. Gene Expression in Adipose Tissues

Quantitative results were obtained by SYBR-Green real-time assay (Euroclone) on total RNA extracted using TRI Reagent^®^ treated with DNase I—RNAase free (Ambion Inc., Foster City, CA, USA) and retro-transcribed using a High Capacity cDNA Reverse Transcription Kit (Ambion, Inc.) as recommended by the manufacturer. Differentially expressed genes were detected by applying a significance threshold on a *t*-test unequal variance.

#### 4.9.2. Gene Expression Profile in Cells

Quantifications of all gene transcripts were performed by real-time retro-transcriptional polymerase chain reaction (Real Time RT-PCR) using a TaqMan^®^ Array Plate 32 (Life Technologies, Paisley, UK,) on Step One Plus^™^ (Applied Biosystems) for the expression of 18S rRNA, GAPDH, with detection as the internal control.

PCR conditions were: 1 cycle at 50 °C for 2 min, followed by exposure at 95 °C for 10 min, then 40 cycles at 95 °C for 15 s, and 60 °C for 1 min. The relative expression of each gene of interest was calculated in relation to the housekeeping gene. The data were only used if the PCR efficiency ranged between 1.85 and 2.0. Template and reverse transcription negative controls were also included in all amplification experiments.

The primers used were:

18S F: AGTACGCACGGGCCGGTACAGTGAACTGCG;
18S R: CGGGTTGGTTTTGATCTGATAAATGCACGC;
GAPDH F: CTTTTGCGTCGCCAG;
GAPDH R: TTGATGGCAACAATATCCAC;
IL1β F: ACAGATGAAGTGCTCCTTCCA;
IL1β R: GTCGGAGATTCGTAGCTGGAT;
TNF-α F: CACTGAAAGCATGATCCGGGACGTGGAGCT;
TNF-α R: TCTTCCCTCTGGGGGCCGATCACTCCAAAG;
*TSG-6* F: CATCTTAATTTACTTATTTCTCTTGCTATG;
*TSG-6* R: TCTGGCTGCCTCTAGCTGCTTGTAAGTTGC;

### 4.10. Statistical Analysis

Data are expressed as means ± standard error of the mean (SEM). The two ways analysis of variance (Anova) and Bonferroni’s post-test were applied using Prism 5 software (GraphPad Software Inc., La Jolla, CA, USA) assuming a *p* value less than 0.05 as the limit of significance.
